# Clearance of fear memory from the hippocampus through neurogenesis by omega-3 fatty acids: a novel preventive strategy for posttraumatic stress disorder?

**DOI:** 10.1186/1751-0759-5-3

**Published:** 2011-02-08

**Authors:** Yutaka Matsuoka

**Affiliations:** 1 Department of Adult Mental Health, National Institute of Mental Health, National Center of Neurology and Psychiatry, Tokyo, Japan; 2 Department of Psychiatry, National Disaster Medical Center, Tokyo; and CREST, Japan Science and Technology Agency, Saitama, Japan

## Abstract

Not only has accidental injury been shown to account for a significant health burden on all populations, regardless of age, sex and geographic region, but patients with accidental injury frequently present with the psychiatric condition of posttraumatic stress disorder (PTSD). Prevention of accident-related PTSD thus represents a potentially important goal. Physicians in the field of psychosomatic medicine and critical care medicine have the opportunity to see injured patients in the immediate aftermath of an accident. This article first briefly reviews the prevalence and associated factors of accident-related PTSD, then focuses on a conceptual model of fear memory and proposes a new, rationally hypothesized translational preventive intervention for PTSD through promoting hippocampal neurogenesis by omega-3 fatty acid supplementation. The results of an open-label pilot trial of injured patients admitted to the intensive care unit suggest that omega-3 fatty acid supplementation immediately after accidental injury can reduce subsequent PTSD symptoms.

## Introduction

Posttraumatic stress disorder (PTSD) is a serious public health problem. Approximately 6.8% of persons in the United States develop PTSD at some time in their lives [1]. PTSD most often develops from traumatic events such as rape, assault and combat, and results far less frequently from experiencing natural disasters and accidents [2]. However, accidental injury is a frequent event and represents a considerable public health burden worldwide. According to the Global Burden of Disease Study, the top three contributors to worldwide burden of disease in the year 2020 are expected to be ischemic heart disease, major depression and motor vehicle accidents (MVAs) [3]. From the Japanese perspective, in 2008 approximately one million individuals were severely injured in MVAs, and recent advances in critical care medicine have increased the number of seriously injured patients who are able to survive their injuries [4]. Over the past decade, increasing attention has been devoted to psychiatric morbidity after accidental injury [5-12] as well as other critical illness requiring treatment in the intensive care unit (ICU) [13]. Indeed, important roles for mental health professionals in general hospitals are the early identification of injured patients who are at risk for developing PTSD and the prevention of the disorder.

It is difficult to eliminate traumatic antecedents altogether, but preventive intervention for PTSD does seem possible. Secondary prevention involves intervening in the aftermath of a traumatic event to forestall the development of PTSD [14]. At the present time, the most well-known and evidence-based secondary preventive intervention for PTSD is cognitive behavioral therapy (CBT). A study by Roberts and colleagues [15] found that trauma-focused CBT within 3 months of a traumatic event appeared to be effective for individuals with traumatic stress symptoms, especially those who met the threshold for a clinical diagnosis. Furthermore, a brochure on bereavement designed as a proactive end-of-life communication strategy was reported to decrease PTSD-related symptoms and symptoms of anxiety and depression among relatives of patients dying in the ICU [16]. Preliminary studies suggest that propranolol [17,18] or cortisol [19,20] can reduce subsequent PTSD, but controlled trials of pharmacologic prevention of PTSD are scarce to date. Although trauma-focused CBT has been demonstrated to be effective, there are few practitioners of psychosomatic medicine working in critical care medicine, and more convenient and evidence-based preventive intervention is desired.

This article provides an overview of the literature on psychiatric morbidity in injured adults admitted to the ICU, with special emphasis placed on PTSD in order to understand the current situation in the field. Following a discussion of the neurobiological mechanism of fear memory, a novel, translational early intervention for preventing PTSD is proposed in which fear memory is minimized through the activation of hippocampal neurogenesis [21].

## Prevalence of PTSD after accidental injury

**Figure 1:**
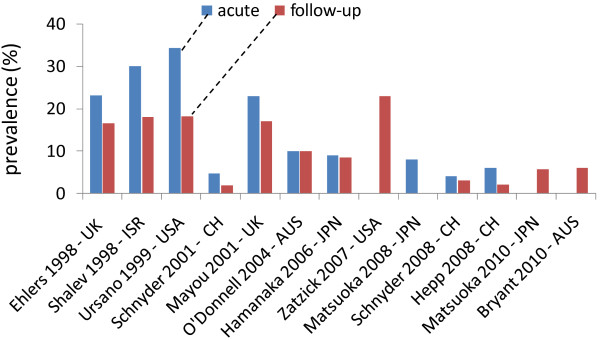
**Prevalence of posttraumatic stress disorder after accidental injury**. Acute and follow-up indicate 0-3 months and 4-12 months after accidental injury, respectively. UK, United Kingdom; ISR, Israel; USA, United States of America; CH, Switzerland; AUS, Australia; JPN, Japan.

Recent studies with strict methodology have shown that accident-related PTSD is fairly common (Figure 1). The prevalence of PTSD determined by structured clinical interviews with injured patients consecutively admitted to the ICU or emergency department ranges from 5-30% at 0-3 months after accidental injury [7,9,10,12,22-24] to 2-23% at 4-12 months after [7,9-11,22-25]. Recent large epidemiological studies have reported a 17-23% point prevalence of questionnaire-estimated clinically significant PTSD symptoms at 4-12 months after accidental injury [5,6]. Comorbidity between PTSD and major depression is also highly prevalent in these injured patients [7,12,26].

It has been pointed out that traumatic brain injury, subsequent traumatization, use of narcotic analgesia, timing of assessment, sample selection, and the role of litigation all have the potential to confound the results for the prevalence of accident-related PTSD [27]. In addition, Schnyder and colleagues found that intercultural differences play an important role in the development of PTSD [11]. Recently, the author and colleagues examined the relation between infant mortality rate and prevalence of PTSD in the reliable cross-country data available [25]. Infant mortality rate is well known to be associated with levels of basic health care, well-developed technology, and medical advances and is also commonly included as part of standard of living evaluations in economics. We showed that infant mortality rate was associated with the prevalence of PTSD and as such, our study findings could provide a plausible explanation for the observed discrepancies seen in the prevalence of PTSD following injury [25].

## Risk factors for accident-related PTSD

Numerous studies have assessed the predictors of accident-related PTSD. Potential risk factors identified in the early aftermath of the accident include increased acute stress symptoms [28,29], female sex [28,29], pre-injury depression [6], ICU admission following the trauma [6], benzodiazepine prescription [6], intentional injury [6], penetrating trauma [28], perceived threat to life [12,26,28], increased heart rate at the time of admission [7,12,30,31], elevated respiration rate on the initial day of injury [30] and intrusive symptoms [10,12]. On the contrary, the risk of subsequent PTSD might be reduced by the use of morphine during trauma care, as demonstrated among US military personnel who experienced combat [32] and individuals injured in accidents [33].

## Consolidation of fear memory

The preclinical approaches to PTSD are examining the mechanisms of memory consolidation and how this consolidation process could be interrupted to prevent the development of trauma-related disorders. An excellent review by Ressler and Mayberg [34] notes that preclinical studies have demonstrated that memories do not immediately become permanent at the time of initial experience. They exist in a labile state for at least a period of hours and possibly days, during which time they become consolidated into more permanent memory. During this consolidation, molecular, synaptic, neurotransmitter and system-level changes occur consecutively [35]. The neural circuitry implicated in fear memory likely involves complex interactions between the hippocampus (which is involved in short-term memory and probably fear of the context of an event), the amygdala (which is involved in conditioned fear response) and the medial prefrontal cortex (which is believed to extinguish the more primitive subcortical response) [36]. The neurocircuitry model of PTSD also implicates the involvement of the amygdala, medial prefrontal cortex and hippocampus [37]. As the hippocampus can process and temporarily store new memory before transferring labile memory to the cortex for permanent storage [38], it has been suggested that during the immediate period after fear training in an animal model and after a traumatic event in human patients, it may be possible to modulate the consolidation of new fear memories in the process of being formed [14].

## Role of hippocampal neurogenesis in memory consolidation

**Figure 2:**
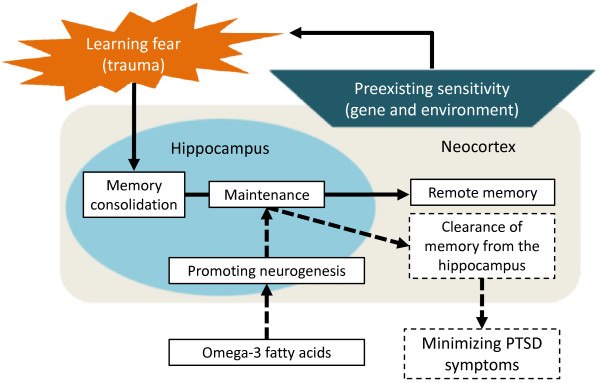
**Schematic illustration of the development of posttraumatic stress disorder (PTSD) focusing on modulating the consolidation of fear memory through neurogenesis by omega-3 fatty acid supplementation**. The strength and regulation of fear memory is affected by many factors both before and after the fearful and traumatic event occurs. Genetic and environmental factors as well as brain function and structure are associated with the risk of such an experience. Acquired memories undergo a period of consolidation, in which they shift from a labile state to a more permanent state. Memories are initially dependent on the hippocampus, but hippocampal dependency progressively decays over time, a process that is associated with a gradual increase in dependency on an extra-hippocampal region such as neocortex (solid arrows and box). We propose that promoting adult neurogenesis by omega-3 fatty acid supplementation early in the transition period might facilitate clearance of fear memory from the hippocampus and consequently minimize PTSD symptoms (dotted arrows and box).

In rodents, primates and humans, the dentate gyrus in the hippocampus is one of the two brain regions with lifelong neurogenesis. Despite the wealth of accumulating data on the characteristics of neurons in newborns, the specific contribution of their generation to memory formation by the hippocampus remains unclear [39]. Recently, Kitamura and colleagues showed that severe impairment of hippocampal neurogenesis attenuated the loss of hippocampus-dependent remote contextual fear memory in mice, while conversely, exercise on a running wheel, which promotes neurogenesis, increased the rate of loss of hippocampus-dependent contextual fear memory [21]. The hippocampus-dependent periods for fear memory are modulated by various conditions. Independent lines of evidence strongly suggest that the level of hippocampal neurogenesis plays a role in determining the hippocampus-dependent period of memory in adult rodents. In short, the level of hippocampal neurogenesis was able to be modulated and was associated with a causal relationship between adult neurogenesis and the hippocampus-dependent period of fear memory. Therefore, it is theoretically possible that promoting adult neurogenesis early in the transition period might facilitate the clearance of fear memory from the hippocampus (Figure 2).

## Modulating consolidation of traumatic memories

According to Pitman's theory of the pathogenesis of PTSD [40], in trauma victims who develop the disorder, the traumatic event stimulates an excess release of stress hormones, which in turn over-consolidates fear memories of the event, which subsequently manifest themselves in the intrusive recollections and re-experiencing symptoms characteristic of PTSD. Fear consolidation can be blocked after training by an antagonist of noradrenergic activation. Such an antagonist is propranolol, a common beta-blocker used for hypertension, and following on from animal research, its effectiveness for the secondary prevention of PTSD has been studied in clinical trials [17,18]. However, as traumatized people are not psychiatric patients, daily life-based intervention for prevention of PTSD is preferable. From my own clinical experience, prophylactic pharmacotherapy targeting subsequent psychiatric illness for injured patients would not be allowed easily.

As much as diet has an impact on cardiovascular health, cancer risk and longevity, it also has an impact on mental health [41]. Adult hippocampal neurogenesis has been directly linked to cognition and mood [42]; therefore, modulating adult hippocampal neurogenesis by diet could emerge as a possible mechanism by which nutrition impacts on mental health. Taken together with Pitman's theory and the conceptual model of fear memory presented herein, it is possible that PTSD can be prevented by facilitating hippocampal neurogenesis in the aftermath of a traumatic event to modulate memory consolidation.

## Omega-3 fatty acids and hippocampal neurogenesis

A growing number of epidemiological studies have suggested an association between mental health and reduced dietary intake of omega-3 fatty acids, essential fatty acids that humans cannot synthesize de novo. Recent clinical trials are supportive of omega-3 fatty acid supplementation in reducing depressive symptoms, although it reduces anxiety symptoms only slightly [43,44]. Based on the animal research to date, omega-3 fatty acids are the most promising candidate for dietary intervention in the aftermath of a traumatic event to facilitate adult hippocampal neurogenesis. Animal studies have revealed that short-term augmentation of dietary omega-3 fatty acids relative to omega-6 fatty acids up-regulated adult neurogenesis [45], and that dietary omega-3 fatty acids elevated levels of brain-derived neurotrophic factor (BDNF) which promotes neuronal survival and growth [46,47]. Further, docosahexaenoic acid (DHA, 22:6*n*-3), a 22-carboned omega-3 fatty acid, promoted the development of hippocampal neurons in vitro by increasing neurite extension and branching [48] as well as the maturation of neurons and hippocampal neurogenesis in adult rats [49]. Venna and colleagues have shown that the increase in newborn hippocampal cells by polyunsaturated fatty acids occurred in parallel with an increase in hippocampal volume and over-expression of BDNF mRNA and protein in the hippocampus [50]. BDNF influences the survival of existing neurons and the growth and differentiation of new neurons, and is also implied in the regulation of various neurotransmitter systems [51,52]. Moreover, BDNF infused directly into the dorsal hippocampus of rats significantly increased the granule cell layer, indicating neurogenesis [53]. Wu and Gomez-Pinilla have indicated that DHA dietary supplementation enhanced the effects of exercise on cognition and BDNF-related synaptic plasticity [47]. Evidence has accumulated that omega-3 fatty acids have an influence on hippocampal neurogenesis by increasing BDNF. In addition, Watanabe and colleagues have revealed that brain fatty acid binding-protein 7 (*Fabp7*) which preferentially binds DHA, plays a significant role in neurogenesis, most likely thorough maintenance of neural stem/progenitor cells [54].

The possible effects of omega-3 fatty acids on brain structures are also highlighted by clinical observation. A significant correlation was found between omega-3 fatty acid consumption and gray matter volume of the amygdala, hippocampus and anterior cingulate gyrus in healthy adults [55]. Conversely, a selective deficit of DHA was reported in the postmortem frontal cortex of patients with depressive disorder [56]. Hippocampal volume appears to be diminished in PTSD in some [57-70] but not all studies [71-77]. The author and colleagues have reported smaller volumes of the amygdala and hippocampus in a cohort of breast cancer survivors experiencing intrusive recollections of traumatic memory, compared to survivors without intrusive recollections [78,79]. Furthermore, a significant negative correlation has been shown between script-driven enhanced emotional memory about MVA and urgent surgery and hippocampal volume in healthy women [80]. Two studies have suggested that hippocampal volume might increase following treatment with antidepressants [81,82]. While the origin of small hippocampal volume is unknown, the result of one twin study suggested that small hippocampal volume might be a familial risk factor for developing PTSD [60]. As well, the nutritional environment, including omega-3 fatty acids, may contribute to hippocampal volume.

## Clinical trial for PTSD prevention by omega-3 fatty acids

**Figure 3:**
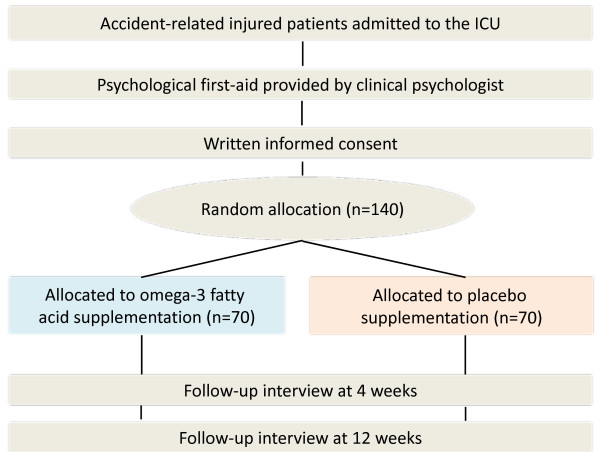
**Flow diagram of the planned progress through the phases of a parallel randomized trial of omega-3 fatty acid supplementation for secondary prevention of PTSD after accidental injury**.

Support for the ability of omega-3 fatty acids to minimize subsequent PTSD symptoms comes from one published but preliminary open trial [83]. The author and colleagues [83] recruited 15 consecutive patients admitted to the ICU at a Japanese general hospital immediately following accidental injury (mostly MVA). Patients received omega-3 fatty acid capsules containing 1,470 mg DHA and 147 mg eicosapentaenoic acid, equivalent to 140 g of grilled cololabis saira 'SANMA in Japanese', daily for 12 weeks. The primary efficacy variable was score on the Clinician-Administered PTSD Scale (CAPS). Omega-3 fatty acid supplementation was well tolerated and resulted in a significantly increased DHA concentration in erythrocytes. Compared with the hypothetical mean in our previous cohort study [84], omega-3 fatty acid supplementation resulted in a significantly reduced mean CAPS total score (11 vs. 25, p = 0.03), and over the 12-week period, only one patient (1/15, 6.7%) developed symptoms consistent with a diagnosis of both PTSD and major depression. Regarding the adherence, significant differences in erythrocyte DHA concentrations were confirmed between weeks 0 and 12 (mean % total fatty acids: 5.9 ± 1.4 vs. 8.4 ± 1.7, p < .001). The author and colleagues also investigated the potential role of BDNF as an underlying mechanism of omega-3 fatty acid action for the prevention of PTSD [85]. Serum BDNF was significantly elevated from weeks 0 to 12 (n = 11, 52.4 ± 16.7 vs. 79.8 ± 13.8, p = 0.001), although it was largely unchanged in the two patients who developed PTSD or major depression during the trial. Change in the serum BDNF between weeks 0 to 12 was significantly larger in the non-distress group than in the distress group, who met the criteria for PTSD or major depression (median, 33.5; range, 8.5-56.0 vs. median, 5.4; range, 4.4-6.4, p = 0.037). Recently, Peters and colleagues [86] have reported excellent work that BDNF infused into the infralimbic medial prefrontal cortex (IL mPFC) reduced conditioned fear, even in the absence of extinction training. And they reported that rats failing to learn extinction showed reduced BDNF in hippocampal inputs to the IL mPFC and that augmenting BDNF in this pathway prevented extinction failure. Hence, our observation would be reasonable. Increasing BDNF activity in hippocampal BDNF may prove to be efficacious intervention for PTSD. Because of the open-label design and the lack of controls, however, no definitive conclusion could be drawn from the trial and we must wait for the results of an adequately powered randomized controlled trial (ClinicalTrials.gov Identifier: NCT00671099) (Figure 3). However, this pilot study has provided promising support for our hypothesis that omega-3 fatty acid supplementation started shortly after accidental injury may be efficacious in attenuating PTSD symptoms.

## Conclusion and perspectives

This review has highlighted the major epidemiologic findings of PTSD and possible nutritional intervention that could be implemented in the aftermath of accidental injury for prevention or amelioration of the disorder. It is now becoming clearer that the modulation of adult hippocampal neurogenesis by diet affects learning, memory, cognition and mood [41]. It is suggested that adult hippocampal neurogenesis may play a role in the periodic clearance of hippocampal memory traces in contextual fear conditioning [21]. Accordingly, adult hippocampal neurogenesis is emerging as a possible mediator of the effect of diet on learning, memory, cognition and mood. Consequently modulating adult hippocampal neurogenesis by omega-3 fatty acid supplementation could be a target of choice to prevent PTSD. Such intervention would likely be acceptable in clinical practice in both mental health and critical care medicine because of its convenience, empirical results in animal studies and less frequent side effects. The paucity of empirical data on nutritional intervention in the immediate aftermath of extreme psychological trauma at present indicates that more controlled trials based on translational research are needed.

## List of abbreviations

BDNF: brain-derived neurotrophic factor; CAPS: Clinician-Administered PTSD Scale; CBT: cognitive behavioral therapy; DHA: docosahexaenoic acid; ICU: intensive care unit; MVA: motor vehicle accident; PTSD: Posttraumatic stress disorder.

## Competing interests

The author declares that they have no competing interests.

## Authors' contributions

The author wrote the manuscript and holds final responsibility for the decision to submit the manuscript for publication.
